# A methodological protocol for the development of a national guideline on perioperative management of gastrointestinal tumors in Germany

**DOI:** 10.1186/s13741-024-00380-0

**Published:** 2024-04-01

**Authors:** M. A. Willis, S. Post, M. Nothacker, M. Follmann, T. Langer, T. O. Vilz

**Affiliations:** 1 Department of General, Visceral, Thorax and Vascular Surgery, University Hospital Bonn, Venusberg-Campus 1, 53127 Bonn, Germany; 2 Department of Surgery, University Hospital Mannheim, Mannheim, Germany; 3 Association of the Scientific Medical Societies e.V., Philipps University Marburg, Marburg, Germany; 4 German Guideline Program in Oncology, German Cancer Society, Berlin, Germany

**Keywords:** Guideline development, Evidence-based patient care, Perioperative management

## Abstract

**Background:**

The success of abdominal cancer surgery depends not only on the surgery itself but is influenced by the overall perioperative management. Given the multitude of perioperative measures and the ever-increasing number of studies on perioperative management, it is difficult to keep track and provide evidence-based perioperative management. The planned guideline on perioperative management will review the existing evidence and derive treatment recommendations.

**Methods:**

The processing of the evidence is carried out by 6 working groups according to an 8-step scheme: after drafting the guideline questions in PICO format (1), a systematic literature search is carried out (2), and the records found are screened by two independent reviewers from the coordination team. Subsequently, the full texts of the potentially relevant articles are made available to the working groups for full text screening (3). All articles to be included are reviewed for methodological quality (4) before summary of findings tables are generated (5). In line with the GRADE approach, confidence in the evidence is assessed (6) before a recommendation is derived from the evidence, using a modified GRADE Evidence to Decision Framework (7). Finally, all recommendations are compiled and agreed within the guideline group (8).

**Discussion:**

Guidelines serve as foundation for therapy decisions in everyday clinical practice and should therefore be based on up-to-date research results. However, while primary studies and systematic reviews are critically reviewed for their methodological quality, the process of guideline development is often not comprehensible. A protocol with predefined methodology should therefore create transparency and strengthen confidence in the recommendations.

**Trial registration:**

The guideline is registered in the AWMF (Association of the Scientific Medical Societies) Guideline Register (088—010OL).

**Supplementary Information:**

The online version contains supplementary material available at 10.1186/s13741-024-00380-0.

## Background

Cancer is a leading cause of death worldwide, accounting for nearly one in six deaths with colorectal cancer being the second leading cause of cancer death (Cancer and Observatory: Cancer Today 2022). However, the steadily increasing number of cancer deaths over the last 10 years paints a distorted picture of malignoma associated mortality. Not every cancer patient can be cured; however, medical progress as well as improved prevention and early detection have caused the age-standardized cancer mortality rate to decline for decades (Krebsinformationsdienst 2022). Colorectal cancer in particular, which account for about 10% of all malignancies (Cancer and Observatory: Cancer Today 2022; Krebsinformationsdienst 2022), can be cured in most cases by extensive surgery. However, the success of the treatment depends not only on the quality of the surgical intervention itself but also on the prevention of postoperative complications and the entire perioperative management (Gamboa et al. 2021; Li et al. 2020).

About 25 years ago, a multimodal, perioperative Fast Track (FT) concept for reducing postoperative complications was developed by Henrik Kehlet (Kehlet 1997), which has been continuously developed since then. The original concept consisted of five recommendations on perioperative measures in colorectal surgery, but new studies on individual aspects of perioperative medicine from a wide range of surgical fields appear regularly, challenging, expanding, or refuting the treatment measures. In order to bundle all available information, the ERAS® (Enhanced Recovery after Surgery) Society was founded in 2001 (ERAS® Society n.d.), which regularly publishes guidelines on the perioperative therapy of various surgical interventions and provides support for implementing this concept in hospitals.

In addition to the ERAS© guidelines, a number of national guidelines have been published that also address aspects of perioperative therapy (Institute and for Health and Care Excellence (NICE) 2020; Work Group of the Clinical Practice Guidelines on Perioperative Care in Major Abdominal Surgery 2016).

Despite the high level of awareness and proven benefits of the FT approach, implementation and maintenance is difficult often resulting in low adherence of only 20–44% (Zelm et al. 2017; Beekum et al. 2020).

As a positive influence on postoperative convalescence has been shown not only for the overall concept but also for the implementation of individual measures, the effectiveness of the individual measures as well as of the overall concept is now to be reviewed again in order to be able to make well-founded recommendations in a new consensus- and evidence-based guideline for perioperative treatment to provide evidence-based care for an accelerated recovery of patients with gastrointestinal malignancies.

## Methods

In Germany, guidelines are classified into four levels (S1, S2e, S2k, S3) based on the underlying methodology and are registered and published in a quality-assured guideline register of the Association of the Scientific Medical Societies (AWMF). In accordance with international methodological requirements, evidence- and formally consensus-based S3 guidelines have the highest methodological quality. They contain predominantly evidence-based recommendations for which a systematic review of the available evidence has been conducted. In addition, they include some recommendations derived from expert opinion on aspects for which there is little published evidence as well as some adaptations of other guidelines where these have been judged to be reliable and of high quality. All recommendations subsequently undergo a formal consensus process (Wissenschaftlichen et al. 2020).

For the development of this guideline on perioperative management of gastrointestinal tumors, a guideline panel was founded in 2019 by the leading professional societies (German Society of General and Visceral Surgery (DGAV) and German Society of Coloproctology (DGK)). This panel is composed of the coordinating team (MAW, SP, TOV), representatives of various professional societies, and selected experts in the field of perioperative care as well as patient representatives. The guideline panel is supported and supervised by representatives of the AWMF (MN) and the German Guideline Program in Oncology (GGPO) (MF and TL).

At a kick-off meeting in September 2020, an agreement was first reached on how to deal with conflicts of interest. Each member of the guideline group disclosed their conflicts of interest, which were then evaluated by the coordination team as either conflict-free or as low, medium, or high. Examples of how the conflicts were rated are given in Table 1, and the consequences are explained:

**Table 1:** Dealing with conflicts of interest

Level of conflict of interest	Example	Consequence
Low	Receiving third-party funding from industry for lectures or authorship	Limited coordinating function
Moderate	Advisory board or consulting activities as well as receipt of third-party funding from industry in a responsible position	Abstain from voting
High	Ownership interest	Abstain from discussing the topic and voting on it

Subsequently, the research questions and patient-related outcome parameters developed by the coordination team were discussed and finally agreed upon by consensus. Furthermore, it was determined which guideline questions should be answered based on expert opinions and which should be evidence-based.

Subsequently, 6 working groups were founded to work on individual aspects of the project, whereby special attention was paid to the composition of the working groups: all groups were assembled interdisciplinary, containing patient representatives as well as known experts in the field. A list of the working groups with the respective PICO questions can be found in Supplement 1.

The evidence-based questions will be addressed by the individual working groups under the supervision of the coordinating team according to an 8-step process developed in line with the GIN-McMaster Guideline Development Checklist (Schünemann et al. 2014).

The guideline is registered in the guideline register of the AWMF (register number 088—010OL, available at https://www.awmf.org/leitlinien/detail/anmeldung/1/ll/088-010OL.html).

### 8-step process of evidence synthesis

**Fig. 1:**
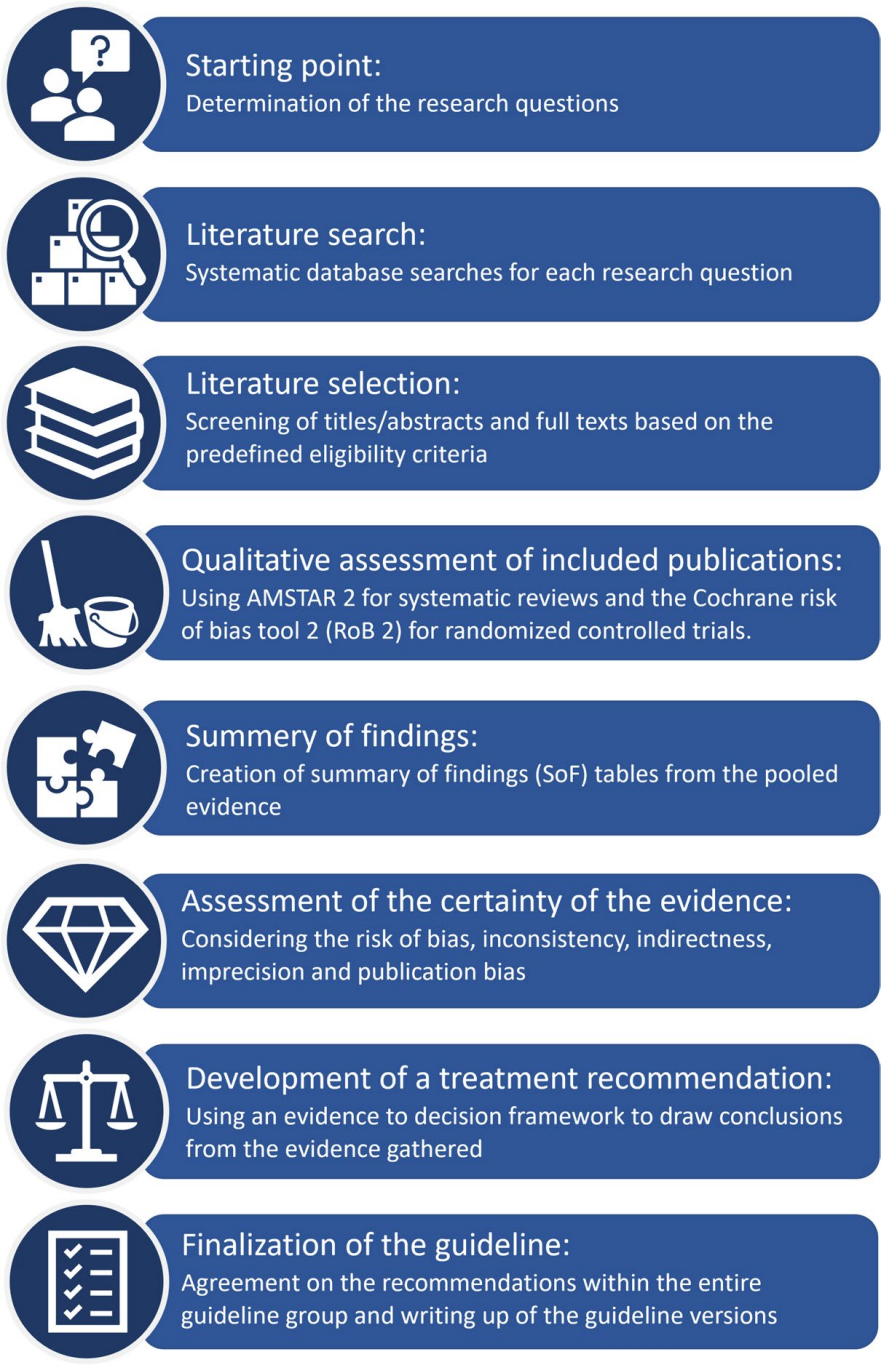
Summary of the 8 steps of the evidence review and recommendation development

Processing of the evidence-based questions follows a multi-stage scheme that defines the methodological procedure. Figure 1 summarizes the 8 steps of the evidence review and recommendation development. Below, the individual steps are explained in detail.

#### Step 1: Starting point

The predefined guideline questions are prepared for evidence-based answering. In some cases, guideline questions are further subdivided as only some aspects are answered in an evidence-based manner or the answer might differ for subgroups (e.g., intraoperative drains depending on the type of surgery performed).

The PICO schema (population, intervention, comparator, outcome) is used to frame the research question, and the predefined outcomes from the kick-off meeting are extended or adapted as necessary.

#### Step 2: Literature search

The strategies for the systematic literature search are being developed by the coordination team. Details on the search strategies, the timing of the literature search, and the databases searched will be presented in the evidence report of the corresponding chapters of the guideline.

If the guideline is not completed within 6 months of the search, updates will be conducted to ensure that no new literature is missing from the guideline.

The literature search is also conducted by the coordination team. First, a search for systematic reviews (SR) containing meta-analyses of randomized controlled trials (RCTs) is done. Afterwards, a search for RCTs is conducted, either as an update search for RCTs published after the baseline review or, if no high-quality review could be found, to conduct an own meta-analysis.

#### Step 3: Literature selection

The title and abstract screening is conducted independently by two members of the coordination team, with a third reviewer resolving any disagreements using the online screening tool Rayyan (Ouzzani et al. 2016). After obtaining the relevant full texts, these are made available to the working groups for full text screening. A pre-selection of the systematic reviews on which the answer to a guideline question could be based is made by two independent working group members on the basis of the pre-established inclusion and exclusion criteria. Disagreements are resolved by discussion and, if necessary, consultation with the guideline coordinator responsible for the working group.

The process of study selection will be presented in the evidence report of the guideline via flow diagrams (Page et al. 2021).

#### Step 4: Qualitative assessment of the included publications

Systematic reviews that meet the inclusion criteria are assessed by the working group according to AMSTAR 2 (Shea et al. 2017). The AMSTAR 2 assessment is used to select one or more reviews to serve as basis for answering the research question. For this purpose, an assessment sheet focusing on the aspects relevant to the guideline was prepared. Among others, an adequate assessment of the risk of bias of the included studies of the meta-analysis is essential for selection as baseline review. Additional RCTs are assessed with the Cochrane Risk of Bias 2 Tool (RoB2) (Sterne et al. 2019), whereby the assessment is performed individually for each of the predefined outcomes.

The AMSTAR 2 and RoB2 assessments of the baseline reviews and included RCTs will be reported in the evidence report of the guideline in the corresponding guideline chapter.

#### Step 5: Summery of findings

For all outcomes predefined in the kick-off meeting, summary of findings tables are created using GRADE proGDT (McMaster University and Evidence Prime 2022). All summary of findings tables will be published in the evidence report of the guideline. If no evidence can be found for a predefined outcome, it is nevertheless listed in the summary of findings table and the missing evidence is marked accordingly.

If a research question is answered based on only one meta-analysis, the pooled data from the meta-analysis is used to create the summary of findings table. However, if additional RCTs that meet the inclusion criteria but are not included in the meta-analysis are identified (for example, because they were published after the search period of the meta-analysis), the following procedure was defined by the coordination team in consultation with the AWMF and the GGPO:

- Option 1: If additional RCTs substantially change the statement of the underlying review, a new meta-analysis is calculated
- Option 2: If additional RCTs are congruent in statement with the underlying review, the following procedures are possible:
  - If the additional RCTs are rather small in size (number of patients) compared to the review or present a high risk of bias, a narrative mention of the new RCTs in the text of the guideline chapter is sufficient and only the review is presented in the summary of findings table
  - If the additional RCTs are large compared to the review and could improve the certainty of evidence according to GRADE, they shall be included in summary of findings table

#### Step 6: Assessment of the certainty of the evidence

The assessment of each outcome in the summary of findings table is made separately by two members of the working group. The following aspects are assessed, leading to an increase or decrease in the confidence of the evidence (Schünemann 2022; Schwenk 2009).

- *Risk of bias:* A high risk of bias or even some concerns about the risk of bias in one or more of the included studies for the outcome may downgrade confidence in the evidence.
- *Inconsistency:* Moderate or considerable heterogeneity between studies that cannot be explained by subgroup analysis may downgrade confidence in the evidence.
- *Indirectness:* Differences between the original PICO question and the included studies regarding population, intervention, comparator, or outcomes may downgrade confidence in the evidence. In particular, when surrogate outcomes are used, transferability must be critically assessed and confidence in the evidence may need to be adjusted.
- *Imprecision:* A small sample size or limited number of events as well as wide confidence intervals are indications of uncertainty about the magnitude of the effect and may lead to a downgrading of confidence in the estimated effect.
- *Large effect:* If the effect is large (RR either > 2.0 or < 0.5 based on consistent evidence from at least 2 studies), this can lead to an increase in confidence in the evidence. In the case of a very large effect (RR either > 5.0 or < 0.2 based on direct evidence with no major threats to validity), even a twofold increase in confidence in the evidence is possible.
- *Plausible confounding:* Circumstances of the studies not taken into account in the meta-analysis, which may lead to an overestimation or underestimation of the effect, may influence the confidence in the evidence.
- *Dose response gradient:* The demonstration of a dose–response relationship can potentially increase the confidence in the evidence.
- Our confidence in the evidence is then expressed as one of four GRADE levels of certainty (see Table 2) (Balshem et al. 2011):

**Table 2:** GRADE levels of certainty of the evidence (Balshem et al. 2011)

Symbol	Quality level	Interpretation
⨁⨁⨁⨁	High	The true effect lies close to that of the estimated effect
⨁⨁⨁◯	Moderate	The true effect is likely to be close to the estimated effect, but there is a possibility that it is substantially different
⨁⨁◯◯	Low	The true effect might be substantially different from the estimated effect
⨁◯◯◯	Very low	The true effect is likely to be substantially different from the estimated effect

#### Step 7: Development of a treatment recommendations (EtD)

To derive treatment recommendations from evidence, the GRADE Evidence to Decision (EtD) framework provides a systematic and transparent approach (Alonso-Coello et al. 2016). We developed a template of an EtD framework adapted to our guideline in which the following aspects of decision-making are taken into account:

- Benefits of the intervention
- Possible harm from the intervention
- Reliability and quality of evidence
- Preferences and acceptability
- Resources and feasibility of the recommendation

According to the EtD principle, the pre-formulated questions are to be answered by the working groups and it is to be stated where the findings come from (evidence or expert opinion). Based on the answers given in the EtD framework, the working group then makes an assessment and derives a conclusion. This conclusion forms the basis for a recommendation text, which is drafted by the working group.

#### Step 8: Finalizing the guideline

Analogous to the consensus-based questions, the individual recommendations developed by the working groups are transferred by the coordinating group to the content management system (CMS) of the GGPO. The CMS offers the possibility for all voting members to review, comment on, or approve the recommendations. The comments received are then discussed again in the working group, and, if necessary, amendments are made to the recommendation or the background text. The basis for any discussion is the EtD framework completed in step 7.

Finally, all recommendations are discussed in a consensus conference with neutral moderation and formal anonymous voting according to the NIH methodology for consensus conferences (National Institutes of Heatlh n.d.). Here, the recommendations are presented by the respective working groups to the whole guideline group, and it is explained how the recommendation was developed using the EtD framework. Possible amendments are discussed and voted on with the aim to reach a consensus. The degree of agreement for a recommendation will be published in the written version of the guideline below the recommendation (> 95% = strong consensus/ > 75–95% = consensus). If no consensus is reached (> 50–75% = majority agreement/ ≤ 50% = no majority agreement), this is also explained.

### Ethics and dissemination

As soon as the guideline is completed, the draft version will be made available to the (professional) public for external review over a period of 6 weeks. In addition, all organizations involved in the development of the guideline including patient representatives will be asked to circulate it to their members for review, including a structured comment form. Any changes resulting from the consultation phase are agreed upon within the guideline group and documented in the guideline report.

Subsequently, the guideline will be approved by the executive boards of all participating professional societies/organizations, and the fulfillment of the S3 requirements are verified before the guideline is published in the AWMF guideline register.

In addition to the long version with background information, an abridged version and a patient version as well as methods report with evidence summaries will also be published. This report—analogous to this protocol—is intended to ensure the transparency of the guideline development process and thus the trustworthiness of the guideline.

All guideline versions as well as the evidence report will be freely accessible via the AWMF and GGPO websites and via an app provided by the GGPO.

## Discussion

For systematic reviews and RCTs, registration of the research projects (RCT for example on ClinicalTrials.gov, systematic reviews on PROSPERO) and the publication of a protocol with predefined methodology are considered as quality indicators. Both the assessment of the risk of bias of individual RCTs (Sterne et al. 2019) and the AMSTAR2 evaluation of systematic reviews (Shea et al. 2017) check whether a protocol with predefined methodology is available. A missing registration will lead to a downgrading of evidence due to risk of bias.

However, particularly for clinical practice guidelines that have a direct impact on daily patient care, it is essential that guidelines are of high quality so that there can be strong confidence in the recommendations made. In Germany, the AWMF guideline register requires registration of each new guideline project or update according to the methodological requirements of the “S” classification, which creates a certain degree of methodological transparency. However, the publication of a full methodological protocol for guidelines is still a rarity and is not included in guideline assessment tools used, such as AGREE II (AGREE Next Steps Consortium 2022).

In the field of perioperative medicine, the ERAS® Society can undoubtedly be considered as a pioneer. The guidelines developed by the ERAS® Society were the first structured set of recommendations on perioperative management. In a methods paper published in 2019 (after dissemination of several recommendations), the ERAS® Society presents its structured approach how they develop new guidelines (Brindle et al. 2020).

Although the recommendations and guidelines from the ERAS® society are already of good methodological quality, we aim for even higher standards for the development of our treatment recommendations for perioperative care, as we want to make comprehensible recommendations based on the highest possible quality of evidence.

Therefore, the literature search for de novo evidence syntheses in our guideline is limited to systematic reviews and RCTs (Level I and II Evidence according to the Levels of Evidence for Therapeutic Studies of the Centre for Evidence-Based Medicine, http://www.cebm.net), whereas the ERAS® guidelines include all publications, regardless of study design. This difference mainly affects the GRADE assessment of evidence certainty, as randomized studies without important limitations (see step 6) have a high quality of evidence, while observational studies without particular strengths are of low-quality evidence as determined in the GRADE-Handbook (Schünemann et al. 2013).

In addition, analogous to the recommendations of the GIN-McMaster checklist for guideline development (Schünemann et al. 2014), we use an evidence to decision framework that specifies the factors that need to be considered when developing a recommendation. This approach is intended to achieve a systematic and transparent way of making well-informed healthcare decisions.

While the original GRADE EtD framework considers seven criteria (benefits and harms of options, values and balance of effects, resources required, cost-effectiveness, equity, acceptability and feasibility) (Moberg et al. 2018), the MAGICapp, for example, also offers a shortened version that considers only four factors (benefits and harms, certainty of evidence, values and preferences, resources and other considerations) (Evidence Ecosystem and Foundation n.d.). In order to focus on the aspects that are crucial for our guideline, we developed our own EtD framework (see step 7). For this, we oriented ourselves on the guidance questions of the GRADEpro GDT online tool (McMaster University and Evidence Prime 2022) as well as on those of the MAGICapp (Evidence Ecosystem and Foundation n.d.). With the exception of cost-effectiveness, all aspects of the original GRADE EtD framework are at least partially covered in our framework. The decision not to include the cost-effectiveness of treatment options in the derivation of recommendations was made by the coordinator team together with the representatives of the AWMF and the GGPO, as we believe that economic interests should not influence patient care.

In contrast to the ERAS guidelines, which are usually developed by a smaller group of experts, our planned guideline will be developed jointly by a large interdisciplinary panel consisting of physicians from different specialities, nurses, physiotherapists, nutritionists, and other professional groups involved in patient care as well as patient representatives. This interdisciplinary composition of the guideline group is intended to better consider different aspects and perspectives and to include them in the formulation of recommendations. Consequently, before the guideline recommendation is released, all recommendations will be discussed in detail in this panel, revised if necessary, and finally agreed upon.

The drawback of the additional methodological effort of our guideline is, however, that this process requires a lot of time and human resources, so that the guideline development process takes about 2 years, whereas ERAS® guidelines are supposed to be developed in a time frame of several months. This lengthy process further limits our guideline, as it is possible that aspects of our guideline may already be outdated at the time of publication, as the latest publications on a topic may not have been considered. Therefore, regular updates of our guideline are necessary. A possible solution to this problem could be the creation of living guidelines, but this would require even more human and time resources.

## Conclusions

In everyday clinical practice, guidelines are the basis of patient care models and serve as orientation for treatment decisions. Therefore, they should be based on current research findings to enable evidence-based therapy. Methodological approaches such as the GRADE assessment of the certainty of evidence or the derivation of recommendations using a structured evidence to decision framework are methods that are intended to increase the quality and impact of our guideline.

However, while primary research articles are critically reviewed for their methodological quality, the guideline development process in many national and international guidelines is not comprehensible.

Nowadays, it is common practice for prospective studies and systematic reviews, and is even considered a quality feature, to register projects and to publish study protocols with predefined methodology. Incomprehensibly, this is still a rarity in guideline projects. This protocol with a predefined methodology, which is based on the recommendations of the GRADE working group, is therefore intended to ensure transparency and increase confidence in the recommendations made in our guideline.

### Supplementary Information

**Additional file 1. **List of the working groups with the respective PICO questions

## Data Availability

Not applicable.

## Abbreviations

AWMF: Association of the Scientific Medical Societies
CMS: Content management system
DGAV: German Society of General and Visceral Surgery
DGK: German Society of Coloproctology
ERAS®: Enhanced Recovery after Surgery
EtD: Evidence to Decision
FT: Fast Track
GGPO: German Guideline Program in Oncology
POMGAT: Perioperative management of gastrointestinal tumors
RCT: Randomized controlled trial
RoB2: Cochrane Risk of Bias 2 Tool
