# Supplementary material for: A methodological protocol for the development of a national guideline on perioperative management of gastrointestinal tumors in Germany
Source: Perioper Med (Lond). 2024 Apr 1;13:25. doi: 10.1186/s13741-024-00380-0 (PMC10983752; doi:10.1186/s13741-024-00380-0)
Supplement: Supplementary file 1 — Additional file 1. List of the working groups with the respective PICO questions [file 13741_2024_380_MOESM1_ESM.docx]

Guideline questions per working group
Guiding questions that will be developed in an evidence-based manner according to the 8-step plan outlined in the protocol are marked in bold.

| WG 1 | Perioperative administration of cardiac drugs   - Beta-blocker - Nitrates - Calcium antagonists - Statins - ACE inhibitors/angiotensin receptor antagonists |
| --- | --- |
|  | Preoperative and intraoperative single dose of corticosteroids |
|  | **PONV prophylaxis**   - **Medicinal** - **Other** |
|  | Sedative premedication   - Benzodiazipine |
| WG 2 | **Prehabilitation** |
|  | Patient education and counselling regarding perioperative interventions /  optimisation options |
|  | Preoperative evaluation and risk assessment |
|  | Hair removal in the surgical area |
|  | Skin disinfection of the operating field |
| WG 3 | Perioperative intravenous antibiotic prophylaxis |
|  | Preoperative bowel preparation   - **Colorectal surgery** - Non-colorectal surgery |
|  | Perioperative selective bowel decontamination (SDD)   - **Upper GI tract** - **Lower GI tract** - Hepatopancreatobiliary |
| WG 4 | Intraoperative insertion of a drain into the surgical field   - Oesophagus - **Stomach** - **Pancreas** - **Liver** - **Colon** - **Rectum** - **Transanal** |
|  | Intraoperative testing of the anastomosis   - Upper GI tract - **Lower GI tract** |
|  | Routine postoperative use of nasogastral tubes   - Oesophagus - **Stomach** - Pancreas - **Liver** - Colorectum |
|  | Leaving the bladder catheter in place beyond the intraoperative phase   - Upper GI tract - Lower GI tract - Hepatopancreatobiliary |

|  | **Supportive therapy using abdominal bandages** |
| --- | --- |
|  | **Postoperative mobilisation** |
|  | Postoperative respiratory therapy |
| WG 5 | **Postoperative pain therapy**   - **Systemic pain therapy** - **Peridural catheter** - **Peripheral regional anaesthesia procedures** |
|  | Prevention and therapy of postoperative gastrointestinal motility disorders   - **Pharmacological prevention and therapy**   - **Prokinetics**   - **Laxatives** - Adjuvant interventions   - Coffee   - Chewing gum   - Electrical stimulation such as acupuncture or transcutaneous electrical nerve stimulation (TENS)   - Other |
| WG 6 | **Care within the framework of a modern multimodal perioperative treatment concept**   - **Upper GI tract** - **Lower GI tract** - **Pancreas** - **Liver** |
|  | Perioperative support by nurses specialised in perioperative interventions |
